# DES2 is a fatty acid Δ11 desaturase capable of synthesizing palmitvaccenic acid in the arbuscular mycorrhizal fungus *Rhizophagus irregularis*

**DOI:** 10.1002/1873-3468.13762

**Published:** 2020-03-03

**Authors:** Henry Cheeld, Govindprasad Bhutada, Frederic Beaudoin, Peter J. Eastmond

**Affiliations:** ^1^ Plant Sciences Department Rothamsted Research Harpenden UK

**Keywords:** arbuscular mycorrhizal fungi, fatty acid desaturase, palmitvaccenic acid, *Rhizophagus irregularis*

## Abstract

Arbuscular mycorrhizal (AM) fungi are oleaginous organisms, and the most abundant fatty acyl moiety usually found in their lipids is palmitvaccenic acid (16:1^Δ11cis^). However, it is not known how this uncommon fatty acid species is made. Here, we have cloned two homologues of lepidopteran fatty acyl‐coenzyme A Δ11 desaturases from the AM fungus *Rhizophagus irregularis*. Both enzymes, DES1 and DES2, are expressed in intraradical mycelium and can complement the unsaturated fatty acid‐requiring auxotrophic growth phenotype of the *Saccharomyces cerevisiae ole1Δ* mutant. DES1 expression leads almost exclusively to oleic acid (18:1^Δ9cis^) production, whereas DES2 expression results in the production of 16:1^Δ11cis^ and vaccenic acid (18:1^Δ11cis^). *DES2* therefore encodes a Δ11 desaturase that is likely to be responsible for the synthesis of 16:1^Δ11cis^ in *R. irregularis*.

## Graphical abstract

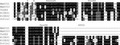

## Abbreviations

**16:1^Δ11cis^**, 11‐cis‐palmitvaccenic acid

**18:1^Δ11cis^**, 11‐cis‐vaccenic acid

**AM**, arbuscular mycorrhizal

**CoA**, coenzyme A

**SCD**, stearoyl‐CoA desaturase

**TEF1**, translational elongation factor EF‐1α

Arbuscular mycorrhiza (AM) is the most common plant–microbe symbiotic association 1. AM fungi are obligate biotrophs and receive organic carbon from their host plants in return for mineral nutrients 1. Lipids are the major carbon currency in the AM fungal mycelium, and they are transported to vesicles and spores where they are stored 2. It was thought that AM fungi most likely synthesize their lipids *de novo* from sugars, which they receive from their host plant 3. However, genomic analysis has suggested that AM fungi are fatty acid auxotrophs 4 and subsequent studies have shown that they rely on their host plant to supply them with long‐chain fatty acyl moieties so that they can make fungal lipids 5, 6, 7, 8. The plant metabolic pathway that supplies fatty acyl moieties to AM fungi has been partially characterized, but it is not yet clear precisely where this pathway ends and those of the fungus begin 5, 6, 7, 8. However, it is currently proposed that long‐chain saturated fatty acyl moieties are most likely being transferred as 2‐monoacylglycerols or free fatty acids 5, 6, 7, 8, 9.

The lipids in many (but not all) AM fungi are dominated by a single molecular species of monounsaturated fatty acid called 11‐cis‐palmitvaccenic acid (16:1^Δ11cis^), which can account for over 70 mol% of the fatty acyl moieties in their spores and is present mainly in the form of triacylglycerols 4, 10, 11, 12. 16:1^Δ11cis^ is unusual in that it contains a double bond at the ω5 (or Δ11) position, and it has been used as a biomarker for arbuscular mycorrhization because it is not found in plants and it is rarely present in other soil microorganisms 10. 16:1^Δ11cis^ has also been used in chemotaxonomy, because it is abundant in many AM fungi (Glomeromycota) but is lacking in certain species of the families Glomeraceae and Gigasporaceae 11.

It is thought that 16:1^Δ11cis^ is made in the intraradical mycelium of AM fungi, but it is not known how 4, 12. The discovery that AM fungi receive fatty acyl moieties from their host plant 5, 6, 7, 8 also raises the possibility that 16:1^Δ11cis^ might be a product of plant metabolism. Understanding how and where 16:1^Δ11cis^ is made is therefore important to define how lipid metabolic pathways function within arbuscular mycorrhiza. Δ11 desaturases have previously been cloned from insects 13, 14 and marine diatoms 15, but we are not aware of any that have been characterized in fungi. The genomes of several AM fungi have now been sequenced, including *Rhizophagus irregularis* 16, which contains 16:1^Δ11cis^ 4. A blastp search (https://www.ncbi.nlm.nih.gov/) of the *R. irregularis* genome using known lepidopteran fatty acyl‐coenzyme A (CoA) Δ11 desaturases 13, 14 revealed two potential homologues (DES1 and DES2). It is problematic to test the function of these genes in AM fungi because they are not amenable to genetic modification. We therefore characterized DES1 and DES2 by heterologous expression in *Saccharomyces cerevisiae* 13 and showed that *DES2* encodes a fungal Δ11 desaturase capable of synthesizing 16:1^Δ11cis^.

## Materials and methods

### Bioinformatic analysis of putative Δ11 desaturases

A blastp search was carried out in NCBI (https://www.ncbi.nlm.nih.gov/) on the *R. irregularis* DAOM197198 genome 16 using functionally characterized Δ11 desaturase sequences from Lepidoptera 13, 14 and marine diatoms 15 as queries. All returned sequences with *E* scores < 0.001 were compiled on a local server and aligned using muscle v3.2 (EMBL‐EBI, Hinxton, Cambridge, UK) 17. Two putative fatty acyl‐CoA desaturases (GenBank accession numbers EXX76018 and EXX69612) were selected for further analysis and were named DES1 and DES2, respectively. The Kyte–Doolittle hydropathy scale with an amino acid window of 19 18 and tmhmm v2.0 (DTU Health Tech, Lyngby, Denmark) (http://www.cbs.dtu.dk/services/TMHMM/) 19 were used for hydropathy analysis and prediction of transmembrane helices (TMHs). signalp v4.0 (DTU Health Tech, Lyngby, Denmark) (http://www.cbs.dtu.dk/services/SignalP) was used for identifying signal peptides at the N and C termini and for distinguishing these from TMHs 20. Searches for conserved domains within protein sequences were carried out using the NCBI Conserved Domain Database (CDD) (https://www.ncbi.nlm.nih.gov/cdd) 21.

### Expression of DES1 and DES2 in *S. cerevisiae*

The open reading frames of *DES1* and *DES2* were codon‐optimized for expression in *S. cerevisiae* by Genscript, synthesized and supplied in the pUC‐57 vector. *DES1* and *DES2* were then excised using *BamHI* and *SalI* restriction sites and ligated into pHEY1 22, for expression under the constitutive translational elongation factor EF‐1α (*TEF1*) promoter. pHEY‐DES1, pHEY‐DES2 and pHEY‐EVC (empty vector) were transformed into *S. cerevisiae* 23 wild‐type (WT) strain DTY‐11a (*MATa*, *leu2‐3*, *leu2‐12*, *trp1–1*, *can1–100*, *ura3–1*, *ade2–1*) and *ole1Δ* knockout strain AMY‐3α (*MATα*, *ole1Δ::LEU2*, *trp1–1*, *can1–100*, *ura3–1*, *ade2–1*) 24, and colonies were selected on synthetic dextrose (SD) minimal medium agar plates lacking uracil. The SD minimal medium used for selection of yeast transformants and culture cultivations consisted of 6.9 g·L^−1^ yeast nitrogen base without amino acids (Formedium, Hunstanton, Norfolk, UK), 1.92 g·L^−1^ yeast synthetic dropout medium supplements minus uracil (Sigma‐Aldrich, St. Louis, MO, USA), 40 mg·L^−1^ of adenine (Sigma‐Aldrich) and 20 g·L^−1^ glucose (Sigma‐Aldrich) as sole carbon source. 10‐mL cultures were grown overnight in SD minimal media to optical density of 0.5–1 at 600 nm and then used to inoculate in 100 mL of SD minimal media to a starting OD600 of 0.1. The 100‐mL cultures were then incubated at 30 °C and shaken at 200 r.p.m. for 72 h. *ole1Δ* cultures were supplemented with 1 mm odd‐ or even‐chain monounsaturated fatty acids (MUFAs), emulsified in 1% (v/v) tergitol (Sigma‐Aldrich). *ole1Δ* was also grown on SD minimal medium agar plates containing fatty acids.

### Lipid extraction and analysis

Cultures were normalized for cell volume based on OD600 measurements and the cells were pelleted by centrifugation at 2400 ***g***, the supernatant discarded, and the pellets frozen in liquid nitrogen and stored at −80 °C. Heptadecanoic acid (17:0) was added to the cell pellets to provide an internal standard (IS). Fatty acid methyl esters (FAMEs) were then prepared from the cell pellets by transmethylation in 1 mL of methanol/toluene/dimethoxypropane/H_2_SO_4_ (66 : 28 : 2 : 1 by volume) at 80 °C for 40 min, before 0.5 mL hexane and 1 mL KCl (0.88% w/v) were added and the contents were vortexed and centrifuged, and the upper hexane phase was transferred to a fresh vial. Extraction with hexane was repeated twice to ensure extraction of all FAMEs and the three extracts pooled. The FAMEs were dried down under N_2_ and reconstituted in 0.5 mL heptane, and 75 µL was taken for analysis by gas chromatography (GC) coupled to mass spectrometry (MS) or flame ionization detector (FID). The position of double bonds in monounsaturated FAMEs was determined by preparing dimethyl disulfide (DMDS) adducts 25. FAMEs (0.1–1 µg) in 50 µL hexane were combined with 5 µL 50 mg·mL^−1^ iodine in diethyl ether and 50 µL DMDS and were vortexed and heated at 40 °C for 15 h. Then, 5 µL 5% (w/v) sodium thiosulfate and 200 µL hexane were added, vortexed and centrifuged to separate the phases. The hexane layer was removed, dried under N_2_ and reconstituted in 50 µL heptane for analysis by GC‐MS. Separation of FAMEs and DMDS adducts was performed by 6890N Network GC System (Agilent Technologies, Santa Clara, CA, USA) fitted with a 30 m × 0.25 mm, 0.25 µm film thickness, HP1‐MS‐UI capillary column (Agilent Technologies). FAME/DMDS adducts (1 µL) were injected (splitless) at 280 °C and He used as the carrier gas (0.6 mL·min^−1^) at a constant flow. The oven program was as follows: 70 °C (1 min), 40 °C·min^−1^ ramp to 150 °C, 4 °C·min^−1^ ramp to 300 °C (2 min), 325 °C (18 min). For FAME/DMDS adduct identification, GC was coupled to a 5975B mass selective detector (Agilent Technologies) with a 3.5‐min solvent delay, on constant scan mode 42–500 *m/z*. The detection and quantification of FAMEs by GC‐FID was also performed, using a DB‐23 capillary column (Agilent Technologies) as described previously 26.

## Results

### Identification of putative ∆11 desaturases

**Fig. 1:**
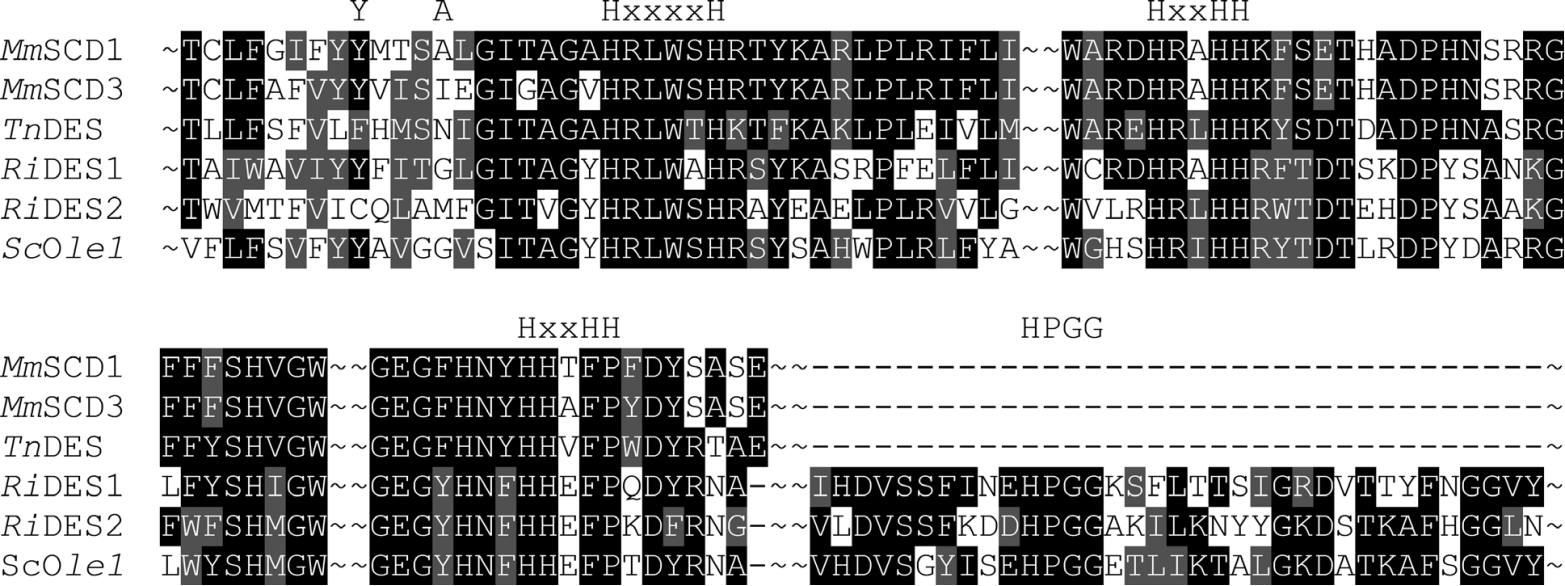
Conserved regions of *Rhizophagus irregularis* DES1 and DES2 aligned with *Trichoplusia ni* Δ11 desaturase and other functionally characterized fatty acyl‐CoA desaturases from *Mus musculus* and *S. cerevisiae*. The three conserved His‐boxes [H(x(n)(H)H] of the desaturase domain and the HPGG box in the C‐terminal cytochrome b5‐like fusion domain are highlighted. Residues Tyr104 and Ala108 that face the substrate binding pocket in *Mm*SCD1 are also highlighted.

To identify candidate ∆11 desaturases from AM fungi, we performed a blastp search of the *R. irregularis* DAOM197198 genome 16 using characterized insect 13, 14 and marine diatom 15 protein sequences. Two genes designated *DES1* and *DES2* (GenBank accession numbers EXX76018 and EXX69612, respectively) were identified that encode proteins that share substantial (> 35%) sequence identity with the archetypal palmitoyl (16:0)‐CoA ∆11 desaturase from *Trichoplusia* *ni* (GenBank accession number AAD03775) 13. Comparison of the amino acid sequences (Fig. 1) revealed that DES1 and DES2 contain a membrane desaturase‐like conserved domain (cl00615) 21 which includes three His‐box motifs, characteristic of desaturases and essential for their catalytic activity 27. In addition, DES1 and DES2 also contain a cytochrome b5‐like haem‐binding conserved domain (cl34968) 21 at their C terminus, featuring a HPGG motif (Fig. 1) that is characteristic of the fusion between desaturases and cytochrome b5 28. Both insect and mammalian fatty acyl‐CoA desaturases lack cytochrome b5‐like C‐terminal extensions, but they are present in fungal counterparts such as the *S. cerevisiae* ∆9 desaturase Ole1p 24, 29. TMHs predicted by the Kyte–Doolittle hydropathy scale 18 and tmhmm 19 were in qualitative agreement (Fig. S1), and both algorithms placed the N and C termini of DES1 and DES2 in the cytosol, consistent with the topology of mammalian stearoyl (18:0)‐CoA desaturases (SCDs) 30. Four TMHs were identified (Fig. S1), which are typical features of membrane‐bound desaturases 30 and are consistent with the crystallographic structures of SCDs 31, 32.

### Expression of *DES1* and *DES2* in *R. irregularis*

To investigate whether *DES1* and *DES2* are expressed in *R. irregularis*, we analysed a RNA‐sequencing data set that includes structures from both asymbiotic and symbiotic stages of the AM fungal life cycle such as germ tubes, runner hyphae, intraradical mycelium, arbuscules, branched absorbing structures and immature and mature spores 33. A search for the corresponding transcripts of *DES1* and *DES2* within this data set revealed that both genes are expressed in all seven AM fungal structures, but *DES2* appears to be the more strongly expressed of the two genes, particularly in intraradical mycelium, arbuscules and spores (Table 1). A desaturase responsible for producing 16:1^Δ11cis^ in *R. irregularis* should be expressed in these structures since this fatty acyl moiety is most abundant in triacylglycerol that accumulates first in lipid droplets that form in the intraradical mycelium proximal to arbuscules 2, 34.

**Table 1:** Transcript abundance of *DES1* and *DES2* in different structures of *Rhizophagus irregularis*. RNA‐sequencing data are derived from Kameoka *et al*. 33 and are expressed as mean log_2_ FPKM (fragments per kilobase of exon per million reads mapped).

Structure	GT	RH	IRM	ARB	BAS	IS	MS
*DES1*	5.88	7.66	6.80	7.90	6.80	8.22	7.22
*DES2*	3.04	6.51	7.99	10.93	9.30	11.68	13.71

ARB, arbuscules; BAS, branched absorbing structures; GT, germ tubes; IRM, intraradical mycelium; IS, immature spores; MS, mature spores; RH, runner hyphae.

### Functional analysis of DES1 and DES2 by expression in *S. cerevisiae*

**Fig. 2:**
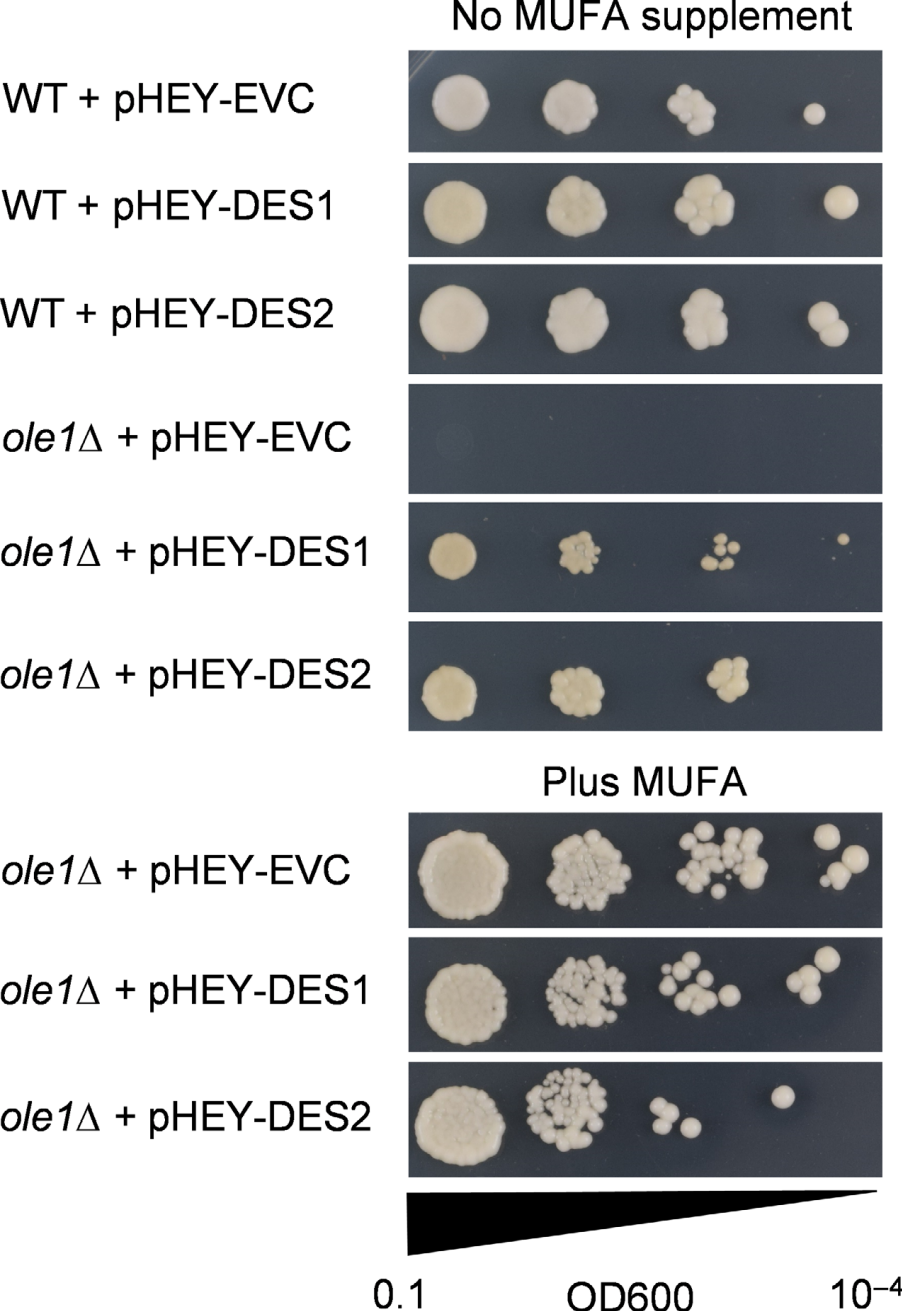
Plate test illustrating the ability of DES1 and DES2 to rescue the unsaturated fatty acid auxotrophic phenotype of *Saccharomyces cerevisiae ole1Δ*. A 0.1‐OD600 culture was successively diluted 10‐fold to 10^−4^, and 2‐µL drops were added to plates with or without a MUFA supplement, using 1 mm 15:1^Δ10cis^. Image was taken after 72 h of growth at 30 °C.

To test the enzymatic function of DES1 and DES2, we transformed WT *S. cerevisiae* and the desaturation‐deficient *ole1Δ* knockout strain 24, 29 with the high‐copy‐number plasmids pHEY‐DES1 and pHEY‐DES2, designed to express the two genes under the control of the strong constitutive *TEF1* promoter 22. The *ole1Δ* strain is completely deficient in fatty acid desaturation and can only grow on media that are supplemented with exogenous long‐chain unsaturated fatty acids 24, 29. A plate test of *ole1Δ* harbouring either pHEY‐DES1 or pHEY‐DES2 showed that cell growth could be rescued by expression of DES1 or DES2 (Fig. 2), suggesting that both proteins can function as desaturases 24, 29.

**Fig. 3:**
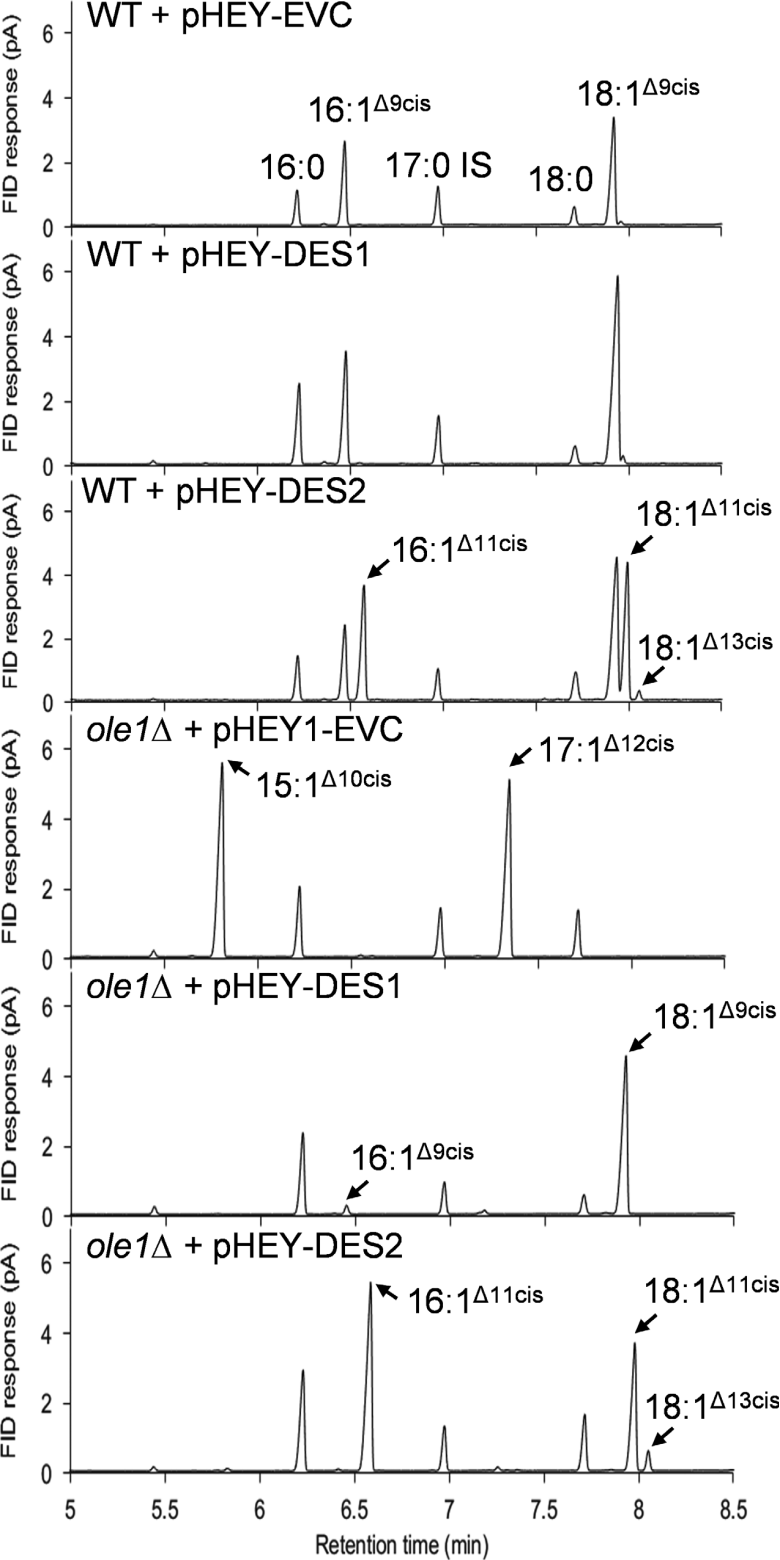
GC‐FID analysis of FAMEs derived from lipid extracts of WT or *ole1Δ* cultures harbouring pHEY vectors, either as EVC or containing DES1 or DES2. For *ole1Δ* + pHEY1‐EVC, an odd‐chain MUFA supplement (15:1^Δ10cis^) was used to complement the *ole1Δ* phenotype and the 17:1^Δ12cis^ is an elongation product of 15:1^Δ10cis^. 17:0 was added to all lipid extracts before transmethylation to provide an IS. The individual GC‐FID traces are representative of three replicates.

Fatty acid methyl ester analysis of lipids from WT *S. cerevisiae* cells 24, 29 expressing DES1 revealed that there was no change in the molecular species that were produced (Fig. 3). However, there was a significant (*P* > 0.05) increase in the relative abundance of oleic acid (18:1^Δ9cis^), as compared to the EVC (Fig. 3; Table S1). By contrast, DES2 expression in WT cells led to the appearance of two major new molecular species of fatty acyl moiety (Fig. 3), which GC‐MS analysis indicated were isomers of 16:1 (*m/z* 268) and 18:1 (*m/z* 296). Further analysis of the double bond positions by extraction of the molecular ions of DMDS adducts 25 revealed the characteristic fragment ions of 16:1^Δ11cis^ (*m/z* 117, 245) and 11‐cis‐vaccenic acid (18:1^Δ11cis^) (*m/z* 145, 245) (Fig. S2). Small amounts of 13‐*cis*‐octadecenoic (18:1^Δ13cis^) (*m/z* 117, 273) were also detected (Fig. 3; Table S1; Fig. S2). Further analysis of the fatty acyl composition of *ole1Δ* cells expressing DES1 or DES2 confirmed that with the substrates that are available, DES1 preferentially produces 18:1^Δ9cis^ over 16:1^Δ9cis^, whereas DES2 produces 16:1^Δ11cis^ and to a lesser extent 18:1^Δ11cis^ (Fig. 3; Table S1).

In WT *S. cerevisiae* cells, trace amounts of 16:1^Δ11cis^ and 18:1^Δ11cis^ were also detected (Table S1). 16:1^Δ11cis^ is known to be a product of 9‐cis‐myristoleic acid (14:1^Δ9cis^) elongation by Elo1p 35, and 18:1^Δ11cis^ is most likely an elongation product of 16:1^Δ9cis^. 16:1^Δ11cis^ elongation is also likely to explain the small amounts of 18:1^Δ13cis^ detected in both WT and *ole1Δ* cells expressing DES2. To test this hypothesis, *ole1Δ* cells expressing DES2 were supplemented with 16:0 or 18:0 free fatty acids to increase the respective amounts of substrate available for desaturation. The addition of 16:0 resulted in a significant increase in 16:1^Δ11cis^ and 18:1^Δ13cis^ (*P* > 0.05), which is consistent with a precursor–product relationship (Table S1). Addition of 18:0 resulted in a significant increase in 18:1^Δ11cis^ (*P* > 0.05), but not in 18:1^Δ13cis^ (Table S1), suggesting that these MUFAs are not products of the same substrate. Taken together, these data suggest that the 18:1^Δ13cis^ is not a direct product of 18:0 desaturation, but of 16:1^Δ11cis^ elongation.

## Discussion

Our data show that *R. irregularis* contains two desaturases that share sequence similarity with fatty acyl‐CoA Δ11 desaturases from Lepidoptera 13, but also possess a cytochrome b5‐like C‐terminal extension characteristic of their fungal Δ9 counterparts 24, 29. DES1 and DES2 are both expressed in the intraradical mycelium where 16:1^Δ11cis^ is thought to be synthesized 4, 12. DES1 and DES2 also both function as desaturases, since they can complement the monounsaturated fatty acid (MUFA)‐deficient phenotype of the *S. cerevisiae ole1Δ* mutant 13, 29. However, analysis of their products shows that DES1 synthesizes 18:1^Δ9cis^ and a little 16:1^Δ9cis^, whereas DES2 synthesizes 16:1^Δ11cis^ and 18:1^Δ11cis^. DES2 activity is therefore most likely to account for the high levels of 16:1^Δ11cis^ that accumulates in *R. irregularis* 4, 11, 12. This finding is also supported by Brands *et al*. 36, who have performed a parallel characterization of DES1 (*Ri*OLE1) and DES2 (*Ri*OLE1‐LIKE).

Desaturases are classified based on their ability to recognize either the ω (methyl) or Δ (carboxyl) end of the fatty acyl moiety for insertion of the double bond 37. The ability of DES1 and DES2 to produce Δ9 and Δ11 fatty acids using substrates with different chain lengths (C16 and C18) suggests that both are front‐end desaturases that count carbon atoms from the carboxyl terminus for insertion of the double bond. The structural basis of chain length specificity has been studied previously in fatty acyl‐CoA desaturases 31. The substrate binding channel of *Mus musculus* SCD1 is capped by Tyr104, which is located on the second transmembrane helix and blocks access of acyl chains longer than C18 31, 38. DES1 also possess Tyr in the corresponding position, while DES2 possesses a less bulky Cys residue (Fig. 1). One helical twist above Tyr104 in *Mm*SCD1, and therefore facing the binding pocket, is Ala108 31. Mutant analysis suggests that when the Ile residue present at this position in *Mm*SCD3 is substituted for Ala, the substrate preference of *Mm*SCD3 changes from C16 to C18 31. Ile has a bulkier side chain than Ala and may therefore shorten the substrate channel 31. DES1 has Gly in this position (Fig. 1), which has a small side chain. DES2 has Met in this position (Fig. 1), which has a slightly larger side chain. The residues occupying these positions might therefore explain why both DES1 and DES2 accept a C18 substrate.

Although 16:1^Δ11cis^ is highly abundant in *R. irregularis,* the levels of 18:1^Δ11cis^ are much lower 4, 12. Given that DES2 can synthesize both MUFAs in *S. cerevisiae*, it is possible that the predominance of 16:1^Δ11cis^ in *R. irregularis* is the result of substrate availability rather than acyl chain length specificity 39. It is thought that *R. irregularis* receives fatty acyl moieties from its host plant that are mainly C16 5, 6, 7, 8, 9 and so this substrate is likely to be most abundant. However, it is also conceivable that 16:1^Δ11cis^ might be preferentially incorporated into triacylglycerol, owing to the activities of lipid assembly and remodelling enzymes that are present in *R. irregularis* but have yet to be characterized 4. *R. irregularis* also contains a comparatively low level of 18:1^Δ9cis^ 4, 12 that is likely to be produced by DES1, given its activity in *S. cerevisiae*. In addition to *R. irregularis*, 16:1^Δ11cis^ is present in many Glomeromycota and putative orthologues of DES2 can also be found in the *R.* *diaphanous*, *R. clarus*, *R. cerebriforme* and *Gigaspora rosea* genomes 40, but not in those of nonmycorrhizal fungi. Interestingly, *G. rosea* is one of the species from the family Gigasporaceae that does not contain 16:1^Δ11cis^ 11, 12. It is therefore possible that *G. rosea* DES2 either has a different activity (i.e. is not a Δ11 desaturase) or is not expressed. At present, it is not known why many Glomeromycota make 16:1^Δ11cis^ and some do not. The identification of DES1 and DES2 may help in future studies to better understand the physiological role of the different molecular species of MUFAs found in AM fungi.

## Author contributions

PJE conceived the research. HC, GB and FB performed the research and analysed the data; and HC and PJE wrote the paper. 

## Supporting information


**Table S1** **.** Fatty acid composition of WT or *ole1Δ* cultures expressing DES1 or DES2.
