# Supplementary material for: DES2 is a fatty acid Δ11 desaturase capable of synthesizing palmitvaccenic acid in the arbuscular mycorrhizal fungus Rhizophagus irregularis
Source: FEBS Lett. 2020 Mar 3;594(11):1770–7. doi: 10.1002/1873-3468.13762 (PMC7317563; doi:10.1002/1873-3468.13762)
Supplement: Supplementary file 1 — Table S1 . Fatty acid composition of WT or ole1Δ cultures expressing DES1 or DES2. [file FEB2-594-1770-s001.pdf]

Supporting information for Cheeld et al. ‘Identification of a Δ11 desaturase from the arbuscular mycorrhizal fungus *Rhizophagus irregularis*’

Table S1. Fatty acid composition of WT or *ole1Δ* cultures expressing DES1 or DES2.

Fatty acid	16:0	$16{:}1^{\Delta 9cis}$	$16{:}1^{\Delta 11cis}$	18:0	$18{:}1^{\Delta 9cis}$	$18{:}1^{\Delta 11cis}$	$18{:}1^{\Delta 13cis}$
WT + pHEY-EVC	12.15 ±0.01	33.61 ±0.02	0.22 ±<0.01	9.27 ±0.02	42.64 ±0.03[a]	2.11 ±0.01	nd
WT + pHEY-DES1	16.72 ±0.01	22.99 ±0.01	0.23 ±<0.01	3.70 ±0.01	54.01 ±0.03[a]	2.34 ±0.01	nd
WT + pHEY-DES2	7.70 ±0.01	15.15 ±0.02	20.90 ±<0.01	5.29 ±<0.01	30.03 ±<0.01	19.86 ±0.03	1.04 ±<0.01
ole1Δ + pHEY-EVC (plus 1mM $15{:}0^{\Delta 10cis}$)	62.80 ±<0.01	nd	nd	37.20 ±<0.01	nd	nd	nd
ole1Δ + pHEY-DES1	28.29 ±0.01	2.64 ±<0.01	nd	6.13 ±<0.01	62.94 ±0.01	nd	nd
ole1Δ + pHEY-DES2	22.29 ±0.01	nd	35.21 ±0.02[b]	10.64 ±<0.01	nd	28.64 ±0.01[d]	3.31 ±<0.01[c]
ole1Δ + pHEY-DES2 (plus 1mM 16:0)	27.66 ±0.03	nd	53.61 ±0.01[b]	4.12 ±0.01	0.14 ±<0.01	8.99 ±0.01	5.45 ±<0.01[c]
ole1Δ + pHEY-DES2 (plus 1mM 18:0)	12.78 ±0.01	nd	30.16 ±0.03	15.24 ±0.01	nd	40.42 ±0.06[d]	1.33 ±<0.01

FAMEs were quantified using GC-FID analysis and their identities were determined by GC-MS analysis of DMDS adducts. nd is not detected. Values are expressed as a percentage of the total and are the mean ±SE of measurements made on cells from three separate cultures. a, b, c and d denote specific pairs of values discussed in the text that are significantly different ($P > 0.05$; two-tailed Student’s t test).

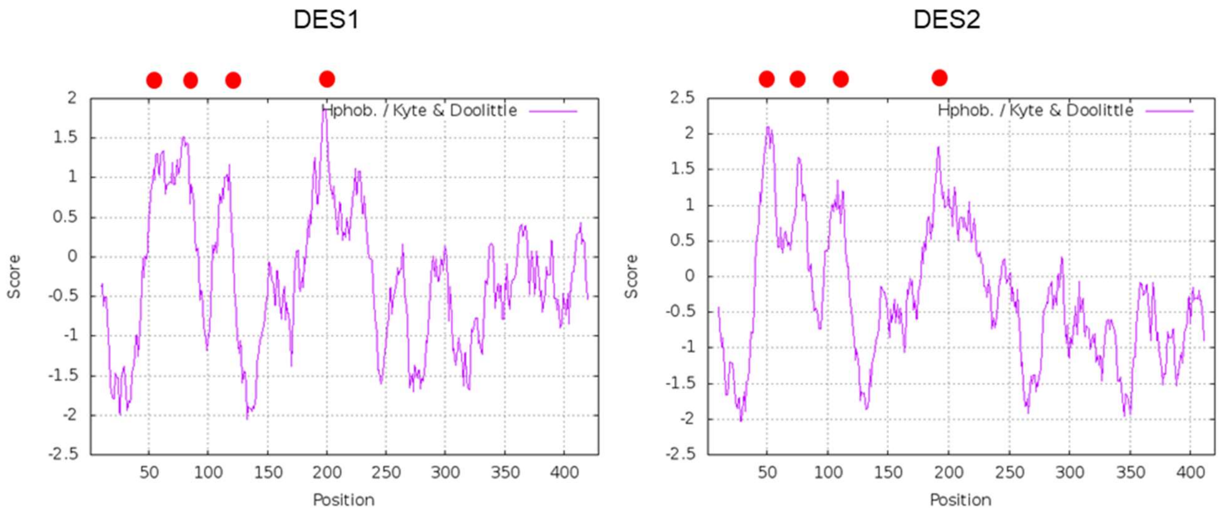

Figure S1. Kyte-Doolittle hydropathy scale with an amino acid window of 19 [18], where regions with a score >0 are considered hydrophobic and scores >1 indicate potential membrane spanning regions. Red dots mark the location of four putative transmembrane helices (TMH) that are also predicted by TMHMM v2.0 [19].

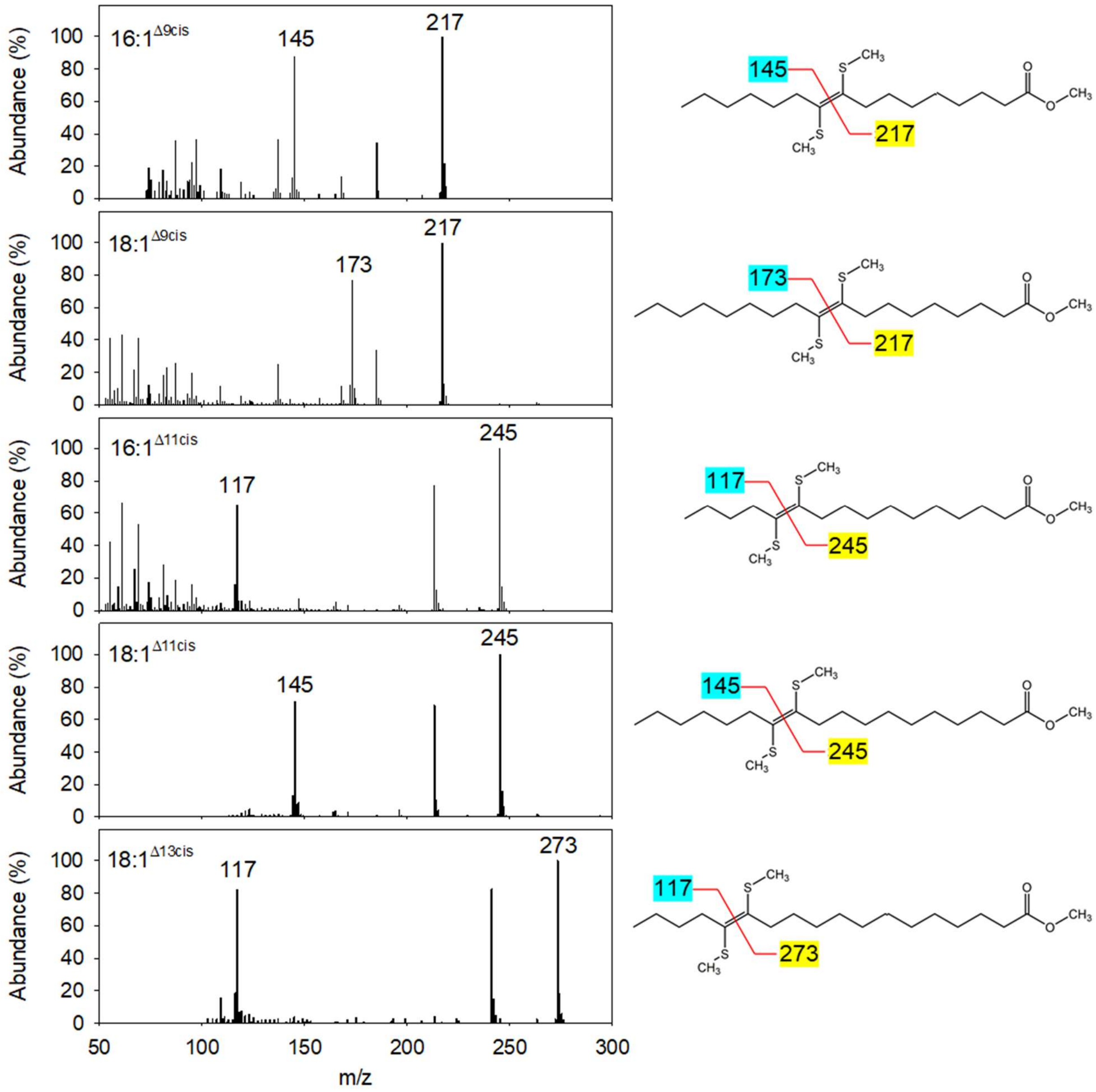

Figure S2. Mass spectra of DMDS FAME adducts. Annotated ions represent diagnostic fragment ions of DMDS adducts used for locating the double bond. Examples of 16:1$^{\Delta9cis}$ and 18:1$^{\Delta9cis}$ from *ole1Δ* + pHEY-DES1 and 16:1$^{\Delta11cis}$, 18:1$^{\Delta11cis}$ and 18:1$^{\Delta13cis}$ from *ole1Δ* + pHEY-DES2.
